# A nutritional protocol and personalized support reduce the cumulative caloric deficit of cardiac surgery patients

**DOI:** 10.1186/cc13617

**Published:** 2014-03-17

**Authors:** E De Waele, K De Bondt, J Czapla, J Nijs, D Nguyen, PM Honoré, H Spapen

**Affiliations:** 1 UniversitairZiekenhuis Brussel, Jette, Belgium

## Introduction

Providing adequate feeding in cardiac surgery patients is gaining importance [1]. The aims were to assess the preoperative nutritional status, to compare postoperative energy needs with effectively delivered amounts, and to evaluate the impact of a dietitian on optimizing postoperative energy balances.

## Methods

A prospective interventional study in adult patients after elective CABG and/or heart valve surgery. The patients' nutritional risk was determined by the NRS 2002 and the Malnutrition Universal Screening Tool (MUST) [2]. A dedicated dietitian managed and assisted the nutritional approach. For each patient, global energy intake (intentional and non-intentional) and balance (difference between energy target and global caloric intake) were calculated daily during and after ICU stay until hospital discharge. The Harris-Benedict equation was used to calculate daily energy requirements. When energy delivery did not reach 60% of calculated needs, a protocol- driven nutritional intervention was initiated.

## Results

Two hundred patients were enrolled during a 10-month period, representing 2,690 study-days. Mean age was 67 ± 11 years. In total, 42.5% of the patients had a NRS 2002 score >3 and were considered to be nutritionally at risk. MUST identified a high, medium, or low risk in respectively 1%, 4% and 95% of subjects. Mean energy requirement was 2,046 ± 347 kcal and mean daily intake 1,452 ± 335 kcal. Sixty-two percent of the caloric need was met during the entire hospital stay. Nutritional intervention was necessary in 52% of cases: oral feeding supplementation for 546 study-days, enteral feeding: 401 days, parenteral feeding: 367 days. This reduced the mean cumulative caloric deficit to 9,085 kcal.

## Conclusion

Close monitoring of caloric intake shouldered by interventions based on a predefined algorithm under supervision of a dedicated nutrition team restricts the caloric deficit in cardiac surgery patients.
